# Measuring the global impact of destructive and illegal fishing on maritime piracy: A spatial analysis

**DOI:** 10.1371/journal.pone.0246835

**Published:** 2021-02-24

**Authors:** Raj M. Desai, George E. Shambaugh

**Affiliations:** 1 Edmund A. Walsh School of Foreign Service, Georgetown University, Washington, District of Columbia, United States of America; 2 Department of Government, Georgetown University, Washington, District of Columbia, United States of America; 3 The Brookings Institution, Washington, District of Columbia, United States of America; Swedish University of Agricultural Sciences and Swedish Institute for the Marine Environment, University of Gothenburg, SWEDEN

## Abstract

Maritime piracy constitutes a major threat to global shipping and international trade. We argue that fishers turn to piracy to smooth expected income losses and to deter illegal foreign fishing fleets. Previous investigations have generally focused on cross-national determinants of the incidence of piracy in territorial waters. These investigations neglect piracy in international waters and ignore its spatial dependence, whereby pirate attacks cluster in certain locations due to neighborhood and spillover effects. We conduct a geographically disaggregated analysis using geo-referenced data of piracy and its covariates between 2005 and 2014. We demonstrate that the incidence of piracy in a particular location is associated with higher catch volumes from high-bycatch and habitat-destroying fishing, even when controlling for conditions in proximate coastal areas. We find, additionally, that illegal, unreported, and unregulated fishing exerts an especially pronounced effect on piracy. These findings highlight the need for anti-piracy solutions beyond enforcement to include the policing of fishing practices that are illegal or are perceived by local fishers in vulnerable coastal areas to be harmful to small-scale fishing economies.

## Introduction

The International Maritime Bureau estimates that maritime piracy costs US$16 billion a year in economic losses due to theft, ransoms, transport delays, increased insurance costs, and anti-piracy protection [1]. Following the surge in piracy off of the Horn of Africa, Somali pirates alone are estimated to have collected some US$340 million in ransoms between 2005 and 2015, prompting a wide range of deterrence interventions, such as naval patrols and armed security aboard vessels sailing through pirate-infested waters [2].

At the same time, there is a growing consensus in both international environmental and security communities that maritime piracy is linked to marine resources [3], and that economic shocks affecting coastal communities play a critical role in explaining piracy [4, 5]. In particular, destructive fishing through habitat degradation and methods producing high bycatch—the incidental harvest of non-targeted fish, protected species, juveniles, and other species without commercial value [6]—generates revenue losses for coastal communities estimated to exceed the total annual contribution of small-scale fisheries to the global economy [7–9]. We posit that destructive and illegal fishing creates expectations of income losses by small-scale fishers, and that the resulting behavioral response increases the likelihood that these fishers will turn to piracy. Moreover, developing an effective understanding of, and a policy response to, maritime piracy requires clarity regarding the relative impact of location-specific factors on its prevalence. Recent scholarship suggests that, as with crime, piracy increases the likelihood of piracy in neighboring maritime areas [10–12]. We propose an approach that explicitly considers the spatial dependence of pirate attacks by analyzing the effects of destructive and illegal fishing practices on the incidence of piracy in all 1° × 1° cells across the ocean. Our study provides one of the first tests of the fishing-piracy nexus on a global scale, and one of the first analyses of the incidence of piracy based on geo-referenced locations rather than cross-national covariates.

## Piracy and maritime livelihoods

Coastal small-scale fisheries account for over half of global seafood capture production and host the majority of the world’s fishers [13]. These small-scale fisheries also provide food security and economic livelihoods for the coastal populations that live in surrounding communities—not only fishers and their families, but up to three times as many others who rely on fisheries for employment and income [14]. Approximately 95-97% of small-scale fishers live in developing countries, many in remote areas where there are few alternative sources of income or employment [9, 15]. It is estimated, further, that almost 20% of small-scale fishers live on less than $1 per day [9].

Coastal economies are vulnerable to a stressors that affect marine ecosystems. Illegal fishing, over-exploitation of related marine resources, climate and ecological change, and ocean pollution all harm the health of local fish populations [16, 17]. More than 60% of all monitored wild fish stocks are fully exploited, and an additional one-fifth is being depleted faster than the replacement rate [18]. These disruptions have disproportionately affected the artisanal fisheries on which small-scale fishers rely, and are a major cause of economic stagnation and impoverishment in coastal communities [19, 20].

In fragile states and conflict-affected areas, moreover, residents of drought-stricken, high-unemployment, or high-poverty areas are frequently targeted for recruitment by violent anti-government groups such as insurgent or terrorist groups, as well as criminal organizations [21–24]. Similarly, pressures on coastal small-scale fishing economies increases the attractiveness of piracy to vulnerable fishermen for two reasons.

First, maritime piracy—alongside other criminal activities—offers a means for those affected by expected earnings losses to supplement their income [25, 26]. Many kinds of illicit activities draw workers from sectors with relevant, transferable skills during hard times [27]. As a result, small-scale fishermen—who have seafaring abilities and navigational knowledge of local waters—are more likely to be recruited by organized pirates than farmers or non-agricultural low-skilled workers [28–31]. The UN Special Representative to Somalia noted that economic hardship, together with a “reduction in pastoralist and maritime resources due to drought and illegal fishing” contributed to the spread of maritime piracy off the Horn of Africa [32, 33]. Indeed, piracy can be highly lucrative; a successful attack in Indonesia typically yields between $900 and $4,000 per pirate from cash and the sale of stolen goods, or up to 30 times the average monthly income for fishermen [34, 35]. Somali pirates can earn between 2 and 5 times the average monthly earnings of fishermen during fishing season, and as much as 135 times this amount during the monsoons [36].

Second, pirate gangs may act as an effective deterrent to foreign industrial or illegal fishing fleets, and thus joining pirate gangs may be a response to fears of foreign exploitation [3, 37, 38]. Local fishers have also been known to hire or join pirate gangs in order to deter foreign industrial fleets from fishing in their territorial waters in the absence of sovereign maritime enforcement [39–41]. In Southeast Asia, as competition for fish and other maritime resources intensified following the Asian Financial Crisis in 1997-1998, increases in industrial fish catch volumes by foreign fleets led to sharp increases in South China Sea pirate attacks organized by residents of villages reliant on small-scale fisheries [42].

## Materials and methods

Our principal contribution lies in explicitly modelling spatial dependence in maritime piracy. Where there is reason to expect piracy to cluster in particular locations, conventional approaches that arbitrarily restrict spatial spillovers to zero or that ignore spatial diffusion can produce estimates that are asymptotically biased. There are three principal reasons to expect spatial dependence among nearby observations. First, spatial proximity can prompt interactions between actors who are likely to be influenced by the behaviors of their neighbors. Second, neighboring units of observations may share characteristics by virtue of the fact that they are proximately located and that actors in those locations, therefore, may behave similarly. Third, factors that influence the behaviors of actors in one location may also influence the behavior of actors in nearby locations. Advances in spatial econometrics have provided techniques for estimating these geographic relationships in regression models [43–45]. To examine the effects of spatial dependence on the risk of maritime piracy, we incorporate spatial lags in the following first-order spatial autoregressive specification, or SAR(1) model:

$$\begin{array}{c} y_{it}=\alpha +\rho Wy_{it}+\beta X+c_{i}+\phi_{t}+\epsilon_{it} \end{array}$$ (1)

$$\begin{array}{c} \text{and}\;X=M+Q+L(1+Q) \end{array}$$ (2)

where *y*_*it*_ is an *NT* × 1 measure of pirate attacks consisting of one observation for every unit in the sample (*i* = 1, …, *N*) for every time period (*t* = 1, …, *T*), **X** denotes a *K*×*NT* matrix of explanatory variables, *β* is a *K* × 1 vector of parameters, *c*_*i*_ is a time-invariant, unit-specific random effect that is uncorrelated with the explanatory variables, *ϕ*_*t*_ is a period-specific intercept, and *ϵ* is a random error, for any given maritime cell *i* in period *t*. Each cell is a 1 × 1° space based on coordinates in the World Geodetic System (WGS 84) equal to 110.6 km of latitude by 111.3 km of longitude at the Equator. In order to accommodate land-based variables that can affect the likelihood of piracy in littoral zones, we expand the domain of analysis to include a coastal “buffer” of 3° inland, or approximately 333 km from the coastline. **M** and **Q** are *K*_*M*_ × *NT* and *K*_*Q*_ × *NT* matrices of marine and land (coastal) based characteristics assumed to influence piracy, respectively (*K*_*M*_+ *K*_*Q*_ = *K*). To control for the differential effect of land vs. maritime factors, we interact **Q** with the *NT* × 1 presence-absence vector L, the components of which take the value of 1 if the cell includes coastal land, and 0 otherwise. Spatial dependence and spatial lags across cells are defined through the symmetric *N* × *N* geographic weighting matrix **W** of non-negative spatial weights. The spatial autoregressive term is *ρ*, and the spatial lag is obtained by multiplying **W** by the vector of observations for the dependent variable. Given the reporting periods of the data sources on which we rely, our consolidated data cover two five-year time periods, 2005-2009, and 2010-2014 (i.e., *T* = 2).

Each element of the weighting matrix **W**, *w*_*ij*_, represents the spatial influence of cell *i* on cell *j*:

$$\begin{array}{c} w_{ij}=\{\begin{array}{cc} d_{ij}^{-\delta } & \text{if}\;d_{ij}\leq \bar{d},\text{for}\;i\neq j\\ 0 & \text{otherwise} \end{array} \end{array}$$ (3)

where *d* is the haversine (great circle) distance between the base point (centroid) coordinates of cell *i* and cell *j*, *δ* is the decay parameter, and $\bar{d}$ is the distance threshold beyond which cells are expected to have zero effect. With *δ* = 1, our matrix relies on a simple inverse-distance weighting, normalized where the (*i*, *j*)^th^ element of the matrix becomes ${\tilde{w}}_{ij}=\frac{w_{ij}}{v}$, and *v* is the largest modulus of the Eigenvalues of **W** (spectral normalization). All matrix values for $d_{ij}>\bar{d}$ take the value of 0, as do diagonal elements since *d*_*ii*_ = 0. We rely on a truncated matrix in which the radius from each cell *i* is limited to $\bar{d}=250\;\text{km}$ between unit centroids, on the assumption that spatial dependence beyond this distance will be negligible. We validate this decision by ensuring that our specifications are robust to weighting matrices expanded to 500 km and 1,000 km thresholds (see S3 and S5 Tables in S1 File). We estimate spatial panel-random effects by quasi-maximum likelihood, using a general-to-specific approach [46, 47]. We start with an unconstrained spatial Durbin error specification in which the spatial lags of the independent variables and the error enter the estimation and proceed to our SAR model based on fit and significance of additional spatial lags, and the log likelihoods of the different estimations (see S2 and S4 Tables in S1 File). The incidence of piracy is calculated as a sum of pirate attacks within a specific geographic cell during each of two five-year periods. We analyze the impact of high-bycatch and habitat-destroying fishing on the incidence of piracy in each cell while controlling for the effects of a number of other mechanisms by which neighboring states would affect the incidence of piracy. These include: state fragility, coastal economic development, climate, and population density, as well as maritime factors such as shipping traffic, ports, and military bases.

### Piracy

The Anti-Shipping Activity Messages (ASAM) database of the US National Geospatial Intelligence Agency is our principal source of data on piracy incidents. These data include the locations of specific hostile acts against ships and mariners. We supplement these data with “Piracy and Armed Robbery” incident reports from the Global Integrated Shipping Information System (GISIS) maintained by the International Maritime Organization. ASAM reports start as early as 1987, while GISIS contains reports beginning in 1995. Given the irregularity of reporting for both ASAM and GISIS prior to 2005, we restrict our data to incidents occurring between January 1, 2005 and December 31, 2014.

**Fig 1:**
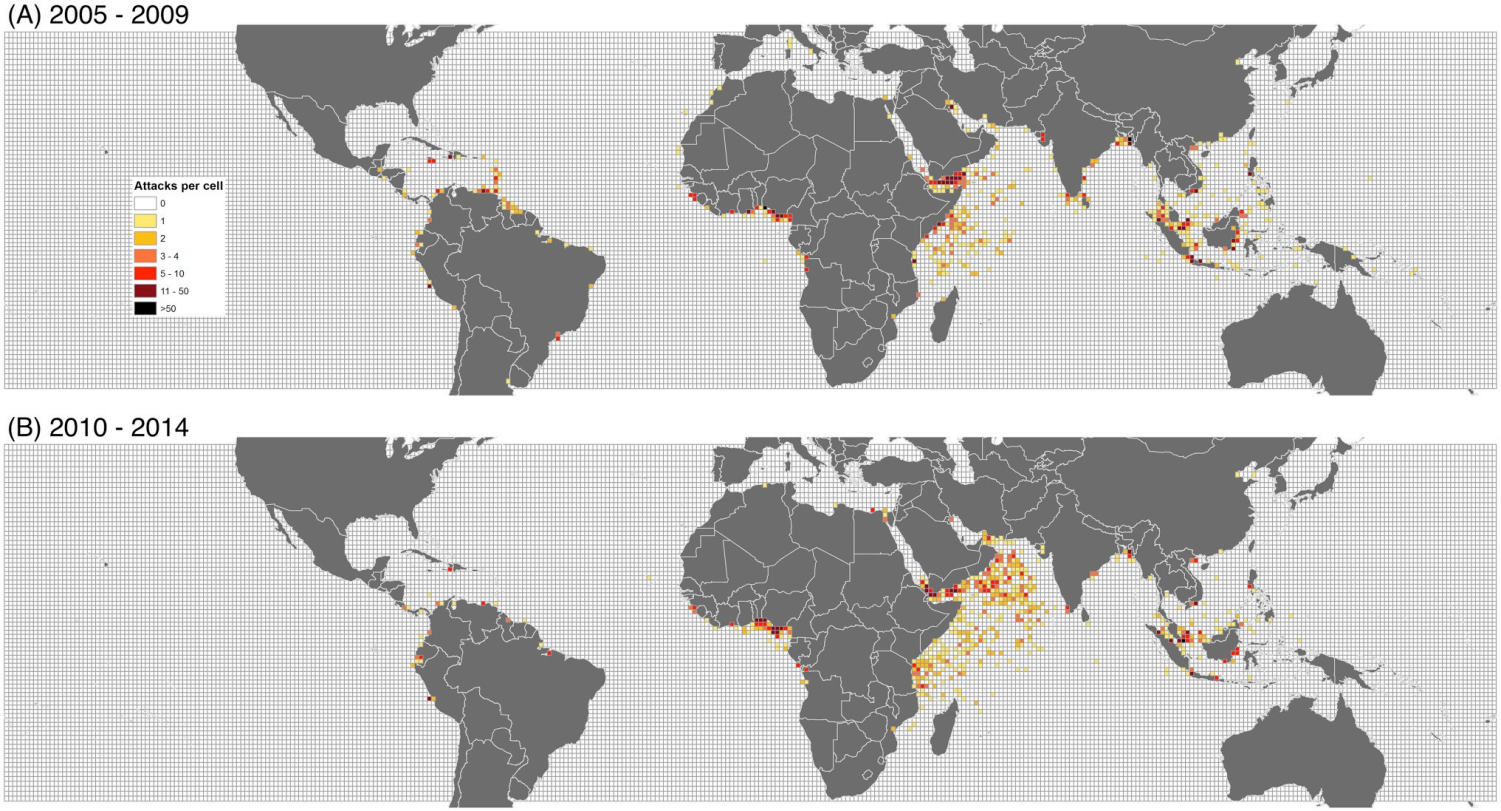
Maritime piracy, 2005-2014. Piracy incidents from the consolidated ASAM-GISIS database, overlaid with a 1° × 1° gridded-cell layer. A: 2005-2009. B: 2010-2014.

The consolidated ASAM-GISIS data are mapped onto the 1° × 1° grid of the world’s oceans between 44 N and 35 S latitudes. All but two reported attacks that occurred globally between 2005 and 2014 took place within this geographic area. Our domain, therefore, consists of 21,836 cells. A total of 3,221 pirate attacks took place in 758 unique cells between 2005 and 2014. For each cell, we have observations for two periods. In 2005-2009, 1,681 attacks were reported in 443 cells; in 2010-2014, 1,540 attacks were reported in 488 cells. Fig 1 shows the total number of pirate-attack locations in the two time periods, as well as the cell grid and the area of coverage. During 2005-2009, 95% of incidents occurred inside a country’s Exclusive Economic Zone (EEZ), the adjacent section of the continental shelf, extending 370 km from the shoreline. Reflecting the diffusion of piracy into international waters, by 2010-2014, the percentage of incidents within EEZs fell to 84%. Our sample, additionally, includes 2,334 cells that cover the 3° inland coastal buffer zones.

A global Moran’s *I* measures the extent to which pirate attacks are clustered, dispersed, or randomly distributed across maritime cells by computing the deviation from the mean number of attacks for each cell [48, 49]. Moran’s *I* values range from –1 (perfect dispersion) to +1 (perfect clustering), while zero corresponds to a random spatial pattern. Spatial clustering is positive and significant within each cell’s 250 km radius (*I* = 0.21, *p*< 0.001), and has increased over the two five-year periods (for 2005-2009, *I* = 0.14, *p*< 0.001; for 2010-2014, *I* = 0.25, *p*<0.001).

If the level of spatial dependency varies across space then the capacity to detect and pinpoint spatial heterogeneity is more desirable. We rely on Getis-Ord analysis to generate a suitable local indicator of spatial autocorrelation. The local Getis-Ord statistic $G_{i}^{*}$ measures the degree of dependence in the count of pirate attacks in cell *i* relative to total attacks across all other cells *j* [50]. The Getis-Ord statistic can discern cluster structures of high- or low-value concentration among local observations, where $G_{i}^{*}>0$ or $G_{i}^{*}<0$, respectively, and is averaged across cells identified as “hot spots”(cells for which $G_{i}^{*}$ is positive and significant, *p* <0.05). Table 1 lists Getis-Ord averages, by FAO-designated fishing area. There are numerous high-value clusters of piracy hot spots, although there is considerable cross-regional variation in local spatial autocorrelation. Between 2005 and 2009, approximately 89% of high-risk cells were found in the Indian Ocean, the West Central Pacific (South China Sea, Philippine Sea, and the waters of the Indonesian archipelago), and the East Central Atlantic (primarily the Gulf of Guinea). By 2010-2014, 95% of hot-spot cells are in those areas—mainly due to the surge in piracy off of the Horn of Africa and Gulf of Aden (Western Indian Ocean region). Although the level of global spatial autocorrelation has risen between the two time periods, aggregate average local spatial autocorrelation fell from 7.2 to 6.0 even as the number of hot spots increased.

**Table 1:** Local spatial autocorrelation of piracy by maritime region.

Ocean (FAO area)	2005–2009		2010–2014	
	*N*	$G_{i}^{*}$	*N*	$G_{i}^{*}$
Western Atlantic (31)	13	5.2 [2.7, 10.7]	4	7.0 [3.7, 11.5]
East Central Atlantic (34)	20	11.7 [2.4, 69.9]	32	9.8 [2.0, 28.2]
Western Indian (51)	86	5.7 [2.0, 42.1]	239	4.7 [2.0, 42.6]
Eastern Indian (57)	24	8.2 [2.0, 90.4]	12	8.4 [2.0, 46.9]
West Central Pacific (71)	32	8.3 [2.0, 27.1]	34	11.0 [2.1, 73.7]
South Eastern Pacific (87)	3	11.8 [2.6, 29.3]	6	6.1 [2.4, 15.4]
Other (37, 41, 47, 61, 77)	5	4.3 [2.6, 7.0]	7	5.2 [2.7, 8.9]
**Full sample**	**183**	**7.2** **[2.0, 90.4]**	**334**	**6.0** **[2.0, 73.7]**

Notes: Table reports the number (*N*) of “hot spot” degree-cells in each maritime region (FAO-designated areas are in parentheses). From these, means of standardized, significant (*p* < 0.05) $G_{i}^{*}$ (in bold) are calculated along with minima/maxima (in brackets) for different time periods and locations. The Getis-Ord statistic for any cell *i* is calculated as $G_{i}^{*}=\frac{\sum_{j=1}^{n}w_{ij}x_{j}}{\sum_{j=1}^{n}x_{j}}$, where *x* is the number of pirate attacks in cell *j* over all *n* cells, and *w*_*ij*_ is the inverse distance between cells *i* and *j* that represents their spatial interrelationship; distances from *i* to *j* are unrestricted. Other FAO areas are those for which *N* ≤ 2: Mediterranean and Black Seas (37), South-Western Atlantic (41), South-East Atlantic (47), North-West Pacific (61), and East-Central Pacific (77). See Fig 2(B) for FAO area locations.

### Destructive fishing

Small-scale fisheries are being increasingly displaced by foreign, industrial fishing fleets and large-scale aquaculture servicing global seafood markets [51, 52]. Extensive industrial fishing can destabilize local food production, livelihoods, and biodiversity. One of the consequences of industrial fishing is high bycatch, which is estimated to constitute over one-third of global fish capture [53, 54]. Bycatch is typically discarded by industrial fleets, directly reducing megafauna populations [55, 56] and indirectly changing trophic dynamics of ocean systems [57, 58]. Non-selective industrial fishing heightens perceptions by local fishers that their livelihoods are at risk [59–61].

**Fig 2:**
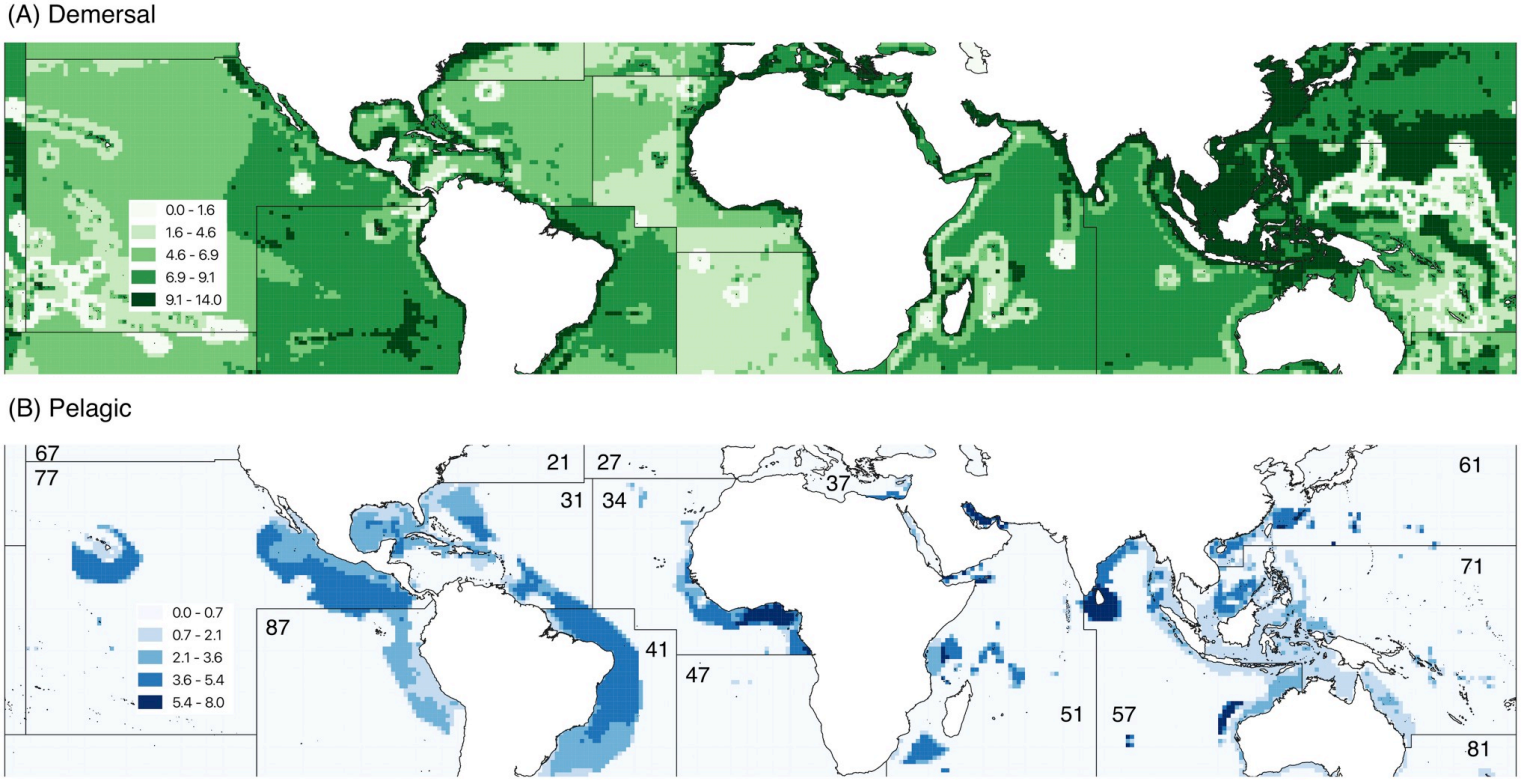
High bycatch and destructive fishing. Estimated total catch, 2005–2014, metric tonnes per degree cell (Ln), by taxonomic group. FAO major fishing areas are labeled in panel (B). FAO major fishing areas reprinted under a CC BY license, with permission from the Food and Agriculture Organization of the United Nations, original copyright 2014. A: High bycatch and habitat-destructive demersal catch. B: High bycatch pelagic catch.

To estimate high bycatch volumes we rely on estimates of global commercial catch classified by type of equipment used and species caught. These data, developed by the *Sea Around Us* project, measure catch from high-bycatch demersal (ocean bottom) and pelagic (non-shore, ranging from ocean surface to benthic zones) fishing [62, 63]. Demersal fishing, additionally, is conducted through habitat-destroying methods such as bottom trawling, dredging, and sometimes through outlawed techniques such as blast or cyanide fishing [64–66]. Reconstructed high-bycatch and destructive demersal and high-bycatch pelagic fishing volumes in metric tonnes per cell for the entire period (2005–2014) are shown in Fig 2. In regression analysis we use five-year totals of fish catch estimates for each cell. We subtract from each cell value the mean total five-year catch estimate of the particular FAO Major Fishing Area in which the cell is located (FAO area boundaries are used to reconstruct raw catch estimates by species and year [67]). Normalizing catch estimates by regional group means allows us to identify the extent to which cell catch estimates deviate from their statistical reference group.

### Illegal, unreported, and unregulated fishing

Deficiencies in the reporting of catches from high bycatch and destructive practices are well known. In particular, relying on reported catch likely ignores the significant volumes of fish caught by fleets that operate in territorial waters without permission, in international waters without flags or other markings, in areas not under the jurisdiction of any regional fisheries management organization, or which do not report (or misreport) catches to relevant authorities [68]. Illegal, unreported, and unregulated (IUU) activity accounts for 15% to 30% of global fish capture, and has been blamed for undermining sustainable fisheries management around the world [69, 70]. Moreover, it is likely that IUU fishing activities and high bycatch and destructive fishing are highly correlated [71].

There are, then, compelling reasons to investigate the relationship between IUU fishing and piracy [72]. We rely on point estimates of IUU catch totals estimated from global fisheries landings in the *Temperate Marine Major Open Data Collection* housed at the University of Tasmania’s Institute for Marine and Antarctic Studies (IMAS). These point estimates are aggregated by each 1° × 1° cell (in metric tonnes) cumulatively for 2005-2009 and 2010-2014.

However, IUU fishing activities may be facilitated by the presence of local piracy, and piracy and IUU fishing may be driven by common factors in proximate states. Organized pirates have been known to extort cash payments from illegal fishing fleets, a form of quasi-rent collection that can be used to finance pirate operations [73]. In addition, piracy could drive away registered industrial fishing fleets and, thus, enable illegal fishers to fill the vacuum. These dynamics make IUU fishing potentially endogenous to piracy, making estimates of their relationship inconsistent.

To address this possible endogeneity we rely on generalized spatial two-stage least squares (GS2SLS) estimation using marine net primary production (NPP), or the rate of photosynthetic carbon assimilation minus the fraction of fixed carbon absorbed by autotrophic and benthic plants, as an instrumental variable. IUU and NPP are expected to be highly correlated since carbon productivity determines the carrying capacity of a marine ecosystem [74]. At the same time, the environmental factors that influence NPP, including light, nutrient abundance, CO_2_, and surface temperatures cannot directly influence, or be influenced by, pirate incidents in any specific maritime location. In sum, NPP fulfills the standard variability and exclusion requirements for an instrumental variable: it affects the potentially endogenous variable IUU catch, and it exerts no direct or confounding effect on the outcome of interest. We rely on carbon production estimates of Moderate Resolution Imaging Spectroradiometer (MODIS) data from NASA’s Terra satellite, based on the Vertically Generalized Production Model (VGPM) [75]. Units of the original data (g C m^−2^ yr^−1^) are aggregated to metric tonnes per degree-cell over each five-year time period.

### Other covariates

Approximately half of the world’s illicit transactions occur in fragile states [76]. In these settings, criminal networks can further challenge state authority, diminish its revenue-raising ability, and erode its judicial capacity. State fragility and collapse are considered precursors of piracy, due to both the absence of state security forces as well as the possibility that pirates act as quasi-state agents in the absence of maritime enforcement [77–79]. We estimate fragility in terms the number of times that the country appeared on the list of fragile states maintained by the Organization for Economic Cooperation and Development (OECD) in each five-year period [80]. States that are more persistently fragile therefore have a higher value for this indicator. When a cell falls entirely within a single country’s EEZ, we apply the fragility value for that country to the cell. When cells include parts of multiple countries or EEZs, we estimate state fragility using a weighted average $F_{i}=\sum_{r=1}^{R}f_{r}s_{r}$ where *f*_*r*_ is the fragility score of the *r*^*th*^ country in EEZ cell *i* which consists of sovereign claims of *r* = 1, …, *R* country EEZs, and *s*_*r*_ is the share of the cell area belonging to the *r*^th^ country’s EEZ claim. For a small number of cells with disputed maritime claims, we take a simple average of the score for all parties that claim the territory. Enforcement capacity, naturally, can be supplemented by multinational anti-piracy naval operations. Within each degree-cell, we count the number of international United States naval bases and coastal US military bases compiled from the Department of Defense [81], along with French and British overseas military bases identified in a European Parliament report [82].

The effect of coastal economies on piracy is debated. Piracy is expected near areas facing unfavorable economic conditions [3, 31, 83–85]. Yet piracy requires functioning markets in which pirates can recruit other members, sell stolen cargo, and acquire financing, weapons, and other equipment needed for forays into territorial and international waters [77, 86]. In cells within 3° inland of the coastline, we include indicators of economic activity levels, drought, and population. We estimate overall levels of economic activity using nighttime luminosity at high resolution based on satellite imagery from the National Oceanic and Atmospheric Administration. We use the mean, uncapped radiance value aggregated for each degree-cell in 2005-2006 and in 2010. We estimate drought using the Standardized Precipitation Evaporation-Transpiration Index (SPEI) from the National Center for Atmospheric Research. The SPEI is considered superior to measures of precipitation-variation as it includes the water retention capacity of vegetation. We examine one-year deviations from the long-run trend; a negative deviation implies drought, a positive deviation flooding. We select minimum values within each cell to identify the severity of drought in every location. Finally, we estimate the total population in each cell using data from the *Gridded Population of the World* project database.

Additional maritime controls include shipping traffic and the location of ports. We count the total number of ports in each cell using the data from World Port Index. We estimate the level of commercial shipping traffic through each cell using mean ship density based on ship locations and movements. Ship movement data are from the National Center for Ecological Analysis and Synthesis. These data are constructed from a sample of vessels representing roughly 11 percent of the merchant ships with >1,000 gross tonnage at sea in 2005, and again in 2013. Definition and measurement of all variables is detailed in S1 Appendix in S1 File, and summary statistics are given in S1 Table in S1 File.

## Results

### Spatial regression results

**Fig 3:**
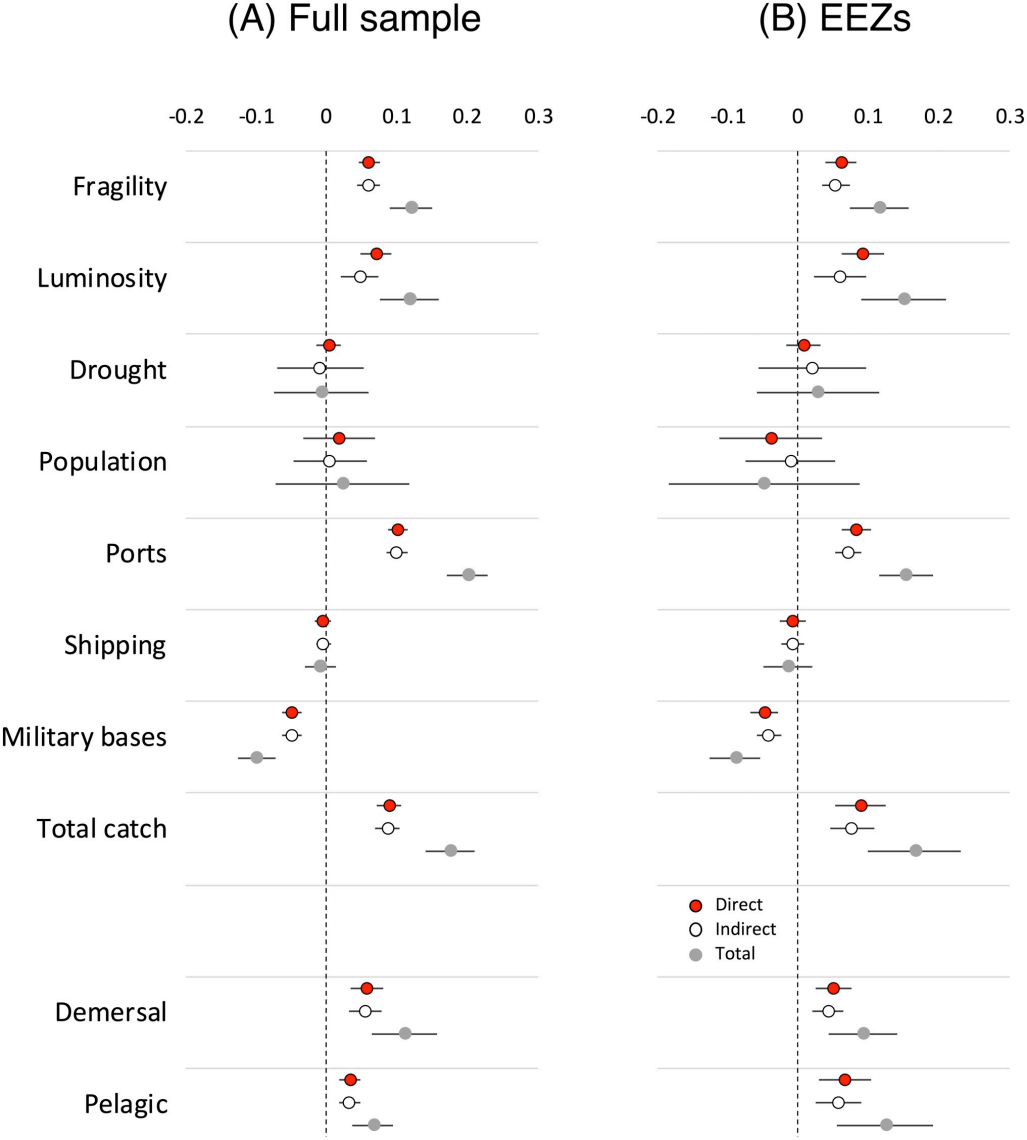
The correlates of piracy. Results are estimated from panel-spatial autoregressive regressions with random effects (see S2 and S4 Tables in S1 File). Dependent variable is number of pirate incidents per degree cell, 2005–2014. Effects of land-based variables (luminosity, drought, and population) are restricted to coastal areas. All variables are standardized. A: Full sample. B: EEZs.

Fig 3 presents the main results from our spatial analysis, decomposing direct effects, indirect or spillover effects, and total effects. Direct effects are the effects of changes in regressors on piracy in the same location. Spatial analysis recognizes that covariates in the present location will affect piracy in neighboring locations which will, in turn, affect piracy in the location under investigation through the spatial dependence parameter *ρ* in Eq 1. Indeed, a change in any covariate will have an indirect effect on the outcome within a specified distance threshold (following the structure of the matrix in Eq 3). These indirect effects, therefore, are global in nature [87]. Total effects comprise the sum of direct and indirect effects. Results in Fig 3 are restricted to ocean (including littoral) cells, excluding entirely land-based cells since piracy cannot take place there.

Effects of eight variables are displayed: state fragility, nighttime luminosity, standardized evaporation-transpiration, population density, number of ports, maritime shipping density, number or military bases, and total habitat-destroying/high bycatch fishing catch differentials with regional means. All values are *z*-standardized by dividing mean differences by standard deviations for each variable. Panel (A) shows the full sample, (B) is restricted to EEZs. Results show consistent, significantly positive effects of harmful fishing on piracy. Higher positive catch differentials increase the risk of piracy in maritime locations whether in EEZs or international waters. A unit increase in the *z*-standardized, log difference between the five-year tonnage of harmful fishing in a given cell above the reference FAO regional average increases the number of pirate attacks by 18%. The effect is due in equal parts to catch volumes in the cell in question and to the spillover effect of piracy in neighboring cells. Similar effects are found when total catch differentials are disaggregated into demersal and pelagic catches.

Governance and enforcement capacity affect piracy as expected. State fragility is associated with increased piracy in EEZs and in international waters. Piracy also has a tendency to cluster around ports. Finally, the presence of American, British, and French military bases deters piracy. Each military base within a cell lowers the number of attacks by 10%.

### Instrumental variable analysis

**Fig 4:**
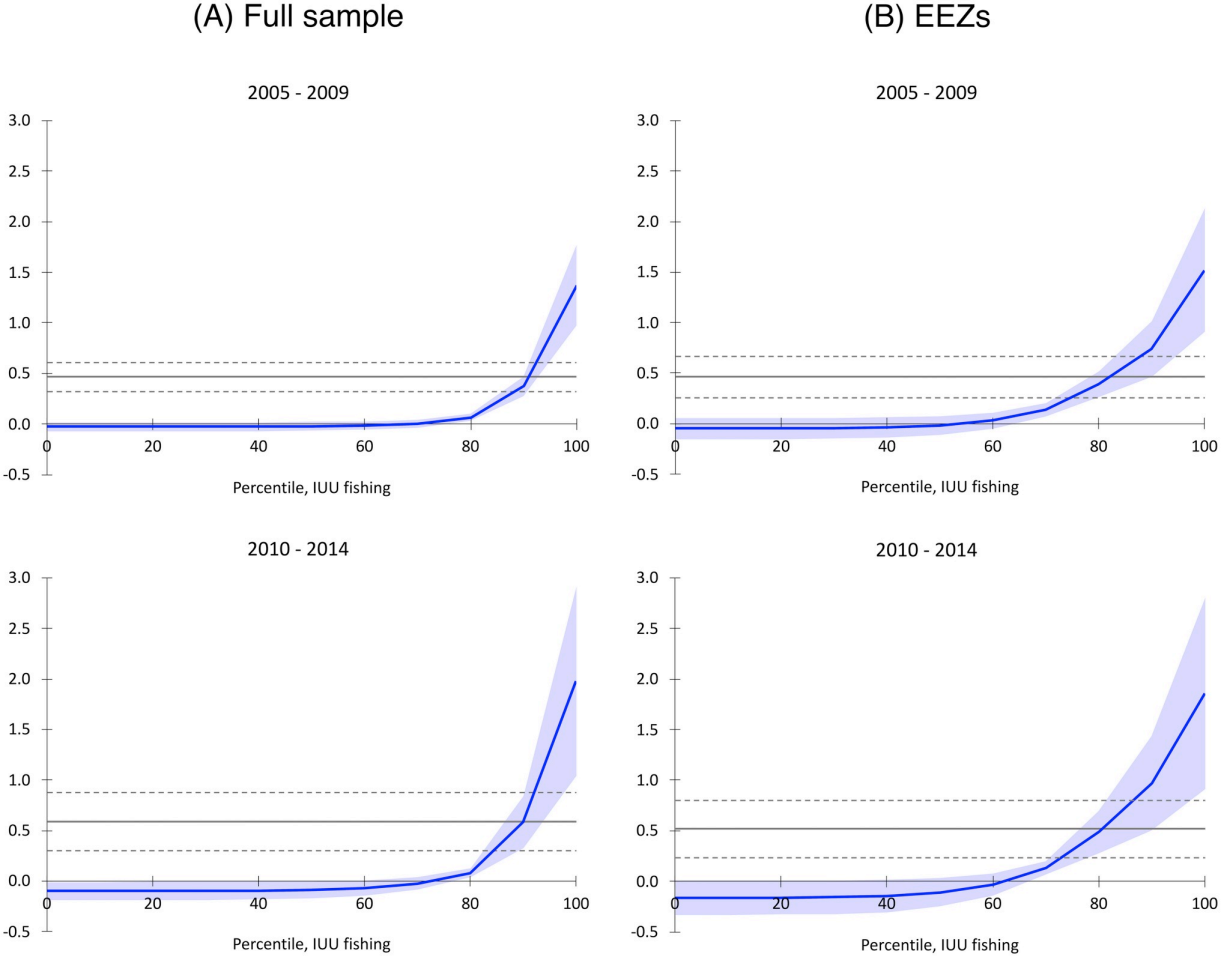
Piracy and illegal, unreported, and unregulated fishing. Results show marginal effects of IUU fishing on the number of pirate attacks per cell over a five-year period, at different percentiles of IUU catch levels, by time period and location, with 95% confidence intervals. Marginal effects are derived from regressions in S6 Table in S1 File. Estimates are reduced-form point effects from generalized spatial two-stage least squares (GS2SLS) incorporating all regressors from Fig 3, with IUU fishing treated as an additional endogenous variable, replacing total catch rates, and instrumented by net primary production. Horizontal lines represent average, reduced-form marginal effects with 95% confidence intervals. A: Full sample. B: EEZs.

Fig 4 presents average and point estimates of the total marginal impact of IUU fishing (instrumented by NPP) on piracy incidence by time period. Our instrumental variable regressions are estimated for each time period separately, using GS2SLS estimation with zero and first-order spatial lags as instruments. We include IUU fish catch as an additional, endogenous covariate, instrumented by zero- and first-order spatial lags of NPP.

Average marginal effects, in all cases, are positively significant. A unit increase in IUU fishing metric tonnes per degree-cell is associated with roughly a 50% increase pirate attacks in both periods. A comparable analysis of IUU fishing on piracy examines cross-national patterns during 1995-2007, and finds that an increase in a measure of IUU catch by two standard deviations, holding all other covariates at their sample means, increases the number of piracy incidents by 72% in a country’s territorial EEZ [72]. In our estimates, a similar change in IUU catch across all maritime locations (correcting for endogeneity and spatial autocorrelation) increases piracy incidents in each degree-cell by 56% in 2005-2009, and by 86% in 2010-2014 (±19% and ±32%, respectively, *p* <0.05). Within EEZs, however, the effect is a 76% increase in piracy incidents in 2005-2009 and a 101% increase in 2010-2014 (±39% and ±47%, respectively, *p* <0.05). The piracy-inducing effect is unequally distributed, with IUU fishing having little marginal impact in the majority of cells, but increasing sharply among the cells accounting for the top quartiles of IUU catch. The locations with the highest risk of piracy are concentrated among those areas with the highest levels of IUU activity.

It has been argued that, as “unreported” and “unregulated” fishing typically describe the practices of some small-scale fisheries, high levels of IUU catch may be associated with greater small-scale fishery capture production [88, 89]. If so, state fragility may also influence IUU fishing, which may be more prevalent in the shadow of weakly-governed states. Most directly, this would be the case for the seas around Yemen and Somalia—two countries in which state collapse may have contributed to both piracy and IUU fishing [90, 91]. To ensure that our results are robust to any such spurious correlation, we rerun the basic spatial instrumental-variables specification, excluding all degree-cells within the Western Indian Ocean FAO Major Fishing Zone (FAO Area 51). This zone comprises the Red Sea from the Suez Canal to the Gulf of Aden, the Persian Gulf, along with the Western and Eastern sides of the Arabian Sea including the territorial waters of Yemen, Eritrea, Somalia, Kenya, Tanzania, and Mozambique, among other states. Restricting analysis to these sub-samples does not change our results (see S6 Table in S1 File).

## Conclusion

Idiographic and cross-county statistical analyses of maritime piracy fail to address errors generated by spatially-induced correlation. Consequently, there is little agreement on the precise spatial determinants of pirate attacks across geography. We examine these spatial correlates directly. Our analyses indicate that fish volumes caught using destructive and high-bycatch methods are robustly associated with piracy. Further, illegal, unreported, and unregulated fishing exerts an especially pronounced effect on piracy, especially in waters that are subject to heavy depredation. These findings highlight the need for solutions that extend beyond anti-piracy enforcement to protecting livelihoods of small-scale fishers, in particular, by policing high bycatch and habitat-destroying practices, and illegal fish capture by foreign industrial fleets. These findings generally support the view that fishers are attractive recruits for piracy, which offers economic rewards as well as the ability to deter perceived threats to small-scale fishing operations.

As expected, piracy is more frequent in waters surrounding fragile states, suggesting a connection between piracy and weak governance. The effect of general economic conditions on piracy is mixed. On one hand, piracy increases near coastal areas with higher overall levels of economic activity. This supports the conclusion that piracy is attracted to areas with market infrastructures, even as pirate incidents increase in the shadow of fragile states. On the other, the incidence of piracy is unaffected by coastal drought or population density. Thus, while piracy is associated with factors that affect maritime livelihoods, it does not appear to be affected by agricultural disruptions or urbanization levels.

Further exploration of the precise longer-term relationship between marine ecosystem stressors and maritime piracy is an avenue for future research. Our evidence suggests a plausible—although by no means absolute—link between catch volume and fish stock status in the long term. Overfished areas, where harmful fishing practices have depleted fish stocks (as appears to be the case in Southeast Asia [92]), may give rise to less resilient coastal communities over time, and more stubborn problems of piracy.

## Supporting information

S1 File (PDF)
