# Supplementary material for: Measuring the global impact of destructive and illegal fishing on maritime piracy: A spatial analysis
Source: PLoS One. 2021 Feb 24;16(2):e0246835. doi: 10.1371/journal.pone.0246835 (PMC7904167; doi:10.1371/journal.pone.0246835)
Supplement: S1 File — (PDF) [file pone.0246835.s001.pdf]

S1 File

S1 Appendix: Data sources and measurement of variables

All units of observation are maritime 1º × 1º cells and, for land-based layers (for variables designated below), 1º × 1º cells within a 3º inland buffer.

Piracy: incident count taken from the Anti-Shipping Activity Messages (ASAM) database of the US National Geospatial Intelligence Agency supplemented with "Piracy and Armed Robbery" incident reports from the Global Integrated Shipping Information System (GISIS) maintained by the International Maritime Organization, occurring between January 1, 2005 and December 31, 2009, and between January 1, 2010 and December 31, 2014.

Fragility: for territorial waters, the number of times the sovereign entity appeared on the OECD's *States of Fragility* reports between 2005-2009, and 2010-2014. See OECD (various).

Luminosity (land-based): Global Radiance-Calibrated Nighttime Lights derived from the Operational Linescan System flown on Defense Meteorological Satellite Program (DMSP) satellites; data are archived at the National Oceanic and Atmospheric Administration (NOAA) National Geophysical Data Center (NGDC). Data in 30 arc-seconds resolution are summed within each degree-cell. Uncapped radiance levels are not reported for all time periods. We use all mean uncapped radiance values available during our time interval, i.e., in 2005-2006 and in 2010.

Drought (land-based): indicator taken from gridded standardized evaporation-transpiration index (SPEI) dataset, available at time scales of 1 to 48 months, at half-degree spatial resolution by the Climate Research Unit, National Center for Atmospheric Research. Data are aggregated to annual values for degree-cells. We examine average one-year deviations from the long-run trend and select the minimum values within each degree-cell for 2005-2009 and 2010-2014.

Population (land-based): population count annual averages for 2005-2009 and 2010-2014 at 15 arc-minute resolution summed within each degree-cell, from *Gridded Population of the World* (version 4), NASA's Socioeconomic Data and Applications Center (SEDAC). In natural logs.

Ports: number of ports summed within each degree-cell over 2005-2009, and 2010-2014.
Location of ports is taken from *World Ports Index* listings, based on criteria established by the US National Geospatial Intelligence Agency.

Shipping: total annual vessel-journeys based on interpolated location information obtained from the National Center for Ecological Analysis and Synthesis based on the Volunteer Observing System (VOS) in 2008, and the VOS and Automatic Identification System (AIS) in 2013, at 6 arc-minute resolution (0.1 decimal degrees), summed by degree-cell. These data are constructed from a sample of vessels representing roughly 11 percent of the merchant ships with > 1,000 gross tonnage at sea. See Halpern, et al. (2008, 2015). Each vessel-journey involves a single vessel entering and exiting the boundaries of the degree cell, with or without stopping. In natural logs.

Military bases: count of number of US, UK, and French military bases within each degree-cell, based on Vine (2015) for US installations, and Rogers and Simon (2009) for UK and French bases.

Pelagic and demersal catch: estimates of destructive and high-bycatch demersal and high-bycatch pelagic fish caught from the *Sea Around Us Project* in metric tons at half-degree resolution, summed by degree cell, in 2008 and again in 2013. Demersal and pelagic catch are categorized according to volume of catch by different types of fishing equipment. In natural logs. See Halpern (2008, 2015).

Illegal, unreported, and unregulated (IUU) catch: estimates of illegal, unreported and unallocated landings based on industrial and non-industrial fishing and associated with certain types of fishing equipment and catch discard rate, in metric tons, from Global Fisheries Landings data (version 4.0) from the *Temperate Marine Major Open Data Collection* housed at the Institute for Marine and Antarctic Studies (IMAS) of the University of Tasmania. Original estimates, mapped to 30 arc-minute resolution, are summed for each degree cell, in natural logs, over 2005-2009 and 2010-2014.

Net primary production (NPP): estimates of carbon-14 based productivity from measurements of sea surface pigment concentrations. We rely on *Moderate Resolution Imaging Spectroradiometer* (MODIS) data from NASA's Terra satellite, based on the Vertically Generalized Production Model (VGPM) (see Behrenfeld and Falkowski 1997). Units of the original data (g C m^{-2}) in 1/6th degree resolution are aggregated to each degree-cell in metric tons, in natural logs, over 2005-2009 and 2010-2014.

S1 Table. Summary statistics

Variable	Mean	Std. Dev.	Min.	Max.
Piracy	0.07	1.09	0	101
Fragility	0.28	1.01	0	5
Luminosity (Ln)	0.17	0.54	0.00	4.92
Drought	-0.15	0.45	-4.40	1.48
Population (Ln)	2.45	4.82	0.00	17.21
Ports	0.09	0.55	0	28
Shipping (Ln)	1.44	1.50	0.00	9.51
Military bases	0.03	0.45	0	20
Total catch (t/cell, Ln)	5.44	2.93	0.00	13.08
Demersal catch (t/cell, Ln)	5.43	2.93	0.00	13.08
Pelagic catch (t/cell, Ln)	0.38	1.01	0.00	7.12
Illegal, unreported, and unregulated catch (t/cell, Ln)	0.26	0.64	0.00	8.38
Net primary production (t C/cell, Ln)	5.10	1.84	0.00	6.28

Notes: $N = 43{,}672$

S2 Table. Panel-spatial regressions

	(1)	(2)	(3)	(4)	(5)	(6)
Fragility	0.045	0.049*	0.059***	0.046	0.049*	0.055***
	(0.024)	(0.023)	(0.008)	(0.024)	(0.023)	(0.008)
Luminosity	0.026	0.014	-0.002	0.010	-0.001	-0.015
	0.054	0.054	0.052	0.054	0.054	0.052
Land	-0.016	-0.019	-0.002	-0.024	-0.027	-0.005
	(0.079)	(0.079)	(0.074)	(0.079)	(0.079)	(0.074)
Land × Luminosity	0.124*	0.137*	0.071	0.142**	0.155**	0.083
	0.054	0.054	0.053	0.054	0.054	0.053
Drought	-0.047	-0.062	-0.055	-0.054	-0.068	-0.056
	(0.144)	(0.143)	(0.143)	(0.143)	(0.143)	(0.143)
Land × Drought	0.047	0.062	0.057	0.050	0.065	0.059
	0.143	0.142	0.143	0.143	0.142	0.143
Population	-0.042	-0.039	-0.032	-0.040	-0.037	-0.031
	(0.091)	(0.091)	(0.090)	(0.091)	(0.091)	(0.090)
Land × Population	0.018	0.012	0.049	0.029	0.023	0.052
	0.097	0.097	0.094	0.097	0.097	0.094
Ports	0.089***	0.089***	0.098***	0.089***	0.089***	0.099***
	(0.008)	(0.008)	(0.007)	(0.008)	(0.007)	(0.007)
Shipping	-0.010	-0.009	-0.005	-0.012	-0.011	-0.006
	0.014	0.013	0.006	0.014	0.013	0.006
Military base	-0.045***	-0.045***	-0.049***	-0.045***	-0.045***	-0.049***
	(0.007)	(0.007)	(0.007)	(0.007)	(0.007)	(0.007)
Total catch	0.173***	0.172***	0.086***			
	(0.016)	(0.016)	(0.008)			
Demersal catch				0.147***	0.146***	0.076***
				(0.016)	(0.016)	(0.009)
Pelagic catch				0.107***	0.104***	0.031***
				(0.016)	(0.016)	(0.007)
**W** × Fragility	0.007	-0.018		0.004	-0.020	
	(0.031)	(0.030)		(0.031)	(0.030)	
**W** × Luminosity	0.913***	0.754***		0.889***	0.737***	
	(0.196)	(0.183)		(0.196)	(0.183)	
**W** × Land	0.486	0.404		0.508	0.426	
	(0.316)	(0.291)		(0.315)	(0.291)	
**W** × Land × Luminosity	-1.145***	-0.980***		-1.125***	-0.965***	
	(0.202)	(0.188)		(0.202)	(0.189)	
**W** × Drought	1.625*	1.347*		1.619*	1.347*	
	(0.645)	(0.577)		(0.645)	(0.577)	
**W** × Land × Drought	-1.624*	-1.342*		-1.614*	-1.338*	
	(0.647)	(0.578)		(0.647)	(0.578)	

**W** × Population	-0.138	-0.104		-0.143	-0.110	
	(0.366)	(0.342)		(0.365)	(0.341)	
**W** × Land × Population	0.330	0.273		0.324	0.266	
	(0.371)	(0.347)		(0.370)	(0.346)	
**W** × Ports	-0.011	-0.026		-0.012	-0.027	
	(0.020)	(0.019)		(0.020)	(0.019)	
**W** × Shipping	0.016	0.011		0.018	0.013	
	(0.017)	(0.016)		(0.017)	(0.016)	
**W** × Military base	0.010	0.018		0.010	0.018	
	(0.019)	(0.018)		(0.019)	(0.018)	
**W** × Total catch	-0.171***	-0.179***				
	(0.025)	(0.024)				
**W** × Demersal catch				-0.144***	-0.152***	
				(0.026)	(0.025)	
**W** × Pelagic catch				-0.111***	-0.110***	
				(0.022)	(0.022)	
**W** × Piracy	0.548***	0.673***	0.551***	0.548***	0.672***	0.549***
	(0.010)	(0.013)	(0.010)	(0.010)	(0.013)	(0.010)
**W** × ϵ		-0.406***			-0.403***	
		(0.039)			(0.039)	
Wald ($p < \chi^2$)	0.000	0.000	0.000	0.000	0.000	0.000
Log(L)	-5.978×10^4	-5.973×10^4	-5.987×10^4	-5.976×10^4	-5.971×10^4	-5.986×10^4
R^2	0.053	0.053	0.038	0.054	0.054	0.039

Notes: Dependent variable is number of pirate incidents per maritime degree-cell during 2005-2009 and 2010-2014. Estimation is using maximum likelihood for spatially-weighted regression with random effects. Spatial-weighting matrix **W** is inverse-distance, spectrally normalized, and truncated to 250 km. All covariates are standardized. Intercepts and FAO fishing-area fixed effects are estimated but not reported. R^2 reported is McFadden's pseudo R^2. Standard errors robust to spatial autocorrelation and heteroskedasticity are in parentheses. N = 43,672. * $p < 0.10$; ** $p < 0.05$; *** $p < 0.01$.

S3 Table. Panel-spatial regressions: Robustness to larger radial truncations

	500km (1) – (3)			1,000km (4) – (6)		
	(1)	(2)	(3)	(4)	(5)	(6)
Fragility	0.027	0.027	0.051***	0.059***	0.060***	0.057***
	(0.018)	(0.018)	(0.008)	(0.014)	(0.014)	(0.008)
Luminosity	0.020	0.019	0.007	0.035	0.038	0.028
	(0.054)	(0.054)	(0.053)	(0.054)	(0.054)	(0.053)
Land	-0.012	-0.012	0.008	-0.010	-0.007	0.008
	0.079	0.079	0.075	0.079	0.079	0.076
Land × Luminosity	0.135*	0.137*	0.074	0.121*	0.120*	0.061
	(0.055)	(0.055)	(0.054)	(0.055)	(0.055)	(0.054)
Drought	-0.037	-0.038	-0.049	-0.001	0.005	-0.032
	0.144	0.144	0.144	0.146	0.146	0.146
Land × Drought	0.033	0.034	0.051	0.002	-0.006	0.036
	(0.144)	(0.144)	(0.144)	(0.146)	(0.146)	(0.146)
Population	-0.050	-0.049	-0.033	-0.022	-0.025	-0.031
	0.092	0.092	0.091	0.093	0.093	0.092
Land × Population	-0.014	-0.016	0.021	-0.023	-0.022	0.018
	(0.098)	(0.098)	(0.095)	(0.098)	(0.098)	(0.096)
Ports	0.092***	0.092***	0.101***	0.093***	0.092***	0.103***
	0.008	0.008	0.007	0.007	0.007	0.007
Shipping	-0.007	-0.007	-0.007	-0.009	-0.006	-0.009
	(0.011)	(0.011)	(0.006)	(0.010)	(0.010)	(0.006)
Military base	-0.047***	-0.047***	-0.051***	-0.051***	-0.050***	-0.053***
	0.007	0.007	0.007	0.007	0.007	0.007
Total catch	0.170***	0.169***	0.090***	0.156***	0.158***	0.094***
	(0.013)	(0.013)	(0.009)	(0.011)	(0.012)	(0.009)
**W** × Fragility	0.017	0.012		-0.055	-0.049	
	(0.030)	(0.030)		(0.030)	(0.033)	
**W** × Luminosity	1.237***	1.184***		2.440***	2.847***	
	(0.335)	(0.331)		(0.595)	(0.656)	
**W** × Land	1.070	1.026		2.358*	2.792*	
	(0.562)	(0.551)		(1.031)	(1.131)	
**W** × Land × Luminosity	-1.579***	-1.522***		-2.989***	-3.431***	
	(0.346)	(0.342)		(0.613)	(0.678)	
**W** × Drought	2.741*	2.606*		4.756*	6.033*	
	(1.165)	(1.137)		(2.096)	(2.366)	
**W** × Land × Drought	-2.742*	-2.605*		-4.774*	-6.053*	
	(1.168)	(1.140)		(2.101)	(2.372)	
**W** × Population	-0.438	-0.433		-1.673	-1.654	
	(0.631)	(0.620)		(1.105)	(1.211)	
**W** × Land × Population	0.803	0.792		2.214*	2.238	
	(0.637)	(0.625)		(1.113)	(1.219)	

**W** × Ports	-0.034	-0.037		-0.031	-0.025	
	(0.032)	(0.031)		(0.051)	(0.057)	
**W** × Shipping	0.014	0.014		0.021	0.023	
	(0.016)	(0.015)		(0.016)	(0.019)	
**W** × Military base	0.039	0.040		0.106*	0.107*	
	(0.029)	(0.029)		(0.047)	(0.052)	
**W** × Total catch	-0.241***	-0.240***		-0.288***	-0.300***	
	(0.029)	(0.028)		(0.035)	(0.040)	
**W** × Piracy	0.760***	0.782***	0.776***	0.902***	0.837***	0.928***
	(0.015)	(0.020)	(0.014)	(0.019)	(0.026)	(0.016)
**W** × ϵ		-0.093			0.398***	
		(0.058)			(0.061)	
Wald ($p < \chi^2$)	0.000	0.000	0.000	0.000	0.000	0.000
Log(L)	-5.993×10^4	-5.992×10^4	-6.004×10^4	-6.023×10^4	-6.022×10^4	-6.035×10^4
R^2	0.057	0.057	0.031	0.056	0.057	0.021

Notes: Dependent variable is number of pirate incidents per maritime degree-cell during 2005-2009 and 2010-2014. Estimation is using maximum likelihood for spatially-weighted regression with random effects. Spatial-weighting matrix **W** is inverse-distance, spectrally normalized, and truncated to 500 km (columns 1 – 3) and 1,000 km (columns 4 – 6). All covariates are standardized. Intercepts and FAO fishing-area fixed effects are estimated but not reported. R^2 reported is McFadden's pseudo R^2. Standard errors robust to spatial autocorrelation and heteroskedasticity are in parentheses. $N = 43{,}672$. * $p < 0.10$; ** $p < 0.05$; *** $p < 0.01$.

S4 Table. Panel-spatial regressions (exclusive economic zones only)

	(1)	(2)	(3)	(4)	(5)	(6)
Fragility	0.048	0.054	0.055***	0.049	0.055	0.052***
	(0.034)	(0.033)	(0.011)	(0.034)	(0.033)	(0.011)
Luminosity	0.020	0.006	-0.007	0.004	-0.010	-0.023
	(0.075)	(0.074)	(0.072)	(0.075)	(0.074)	(0.072)
Land	-0.001	-0.009	0.011	-0.008	-0.016	0.014
	(0.110)	(0.109)	(0.104)	(0.110)	(0.109)	(0.104)
Land × Luminosity	0.129	0.144	0.083	0.148*	0.163*	0.097
	(0.075)	(0.075)	(0.074)	(0.075)	(0.075)	(0.074)
Drought	-0.079	-0.101	-0.089	-0.084	-0.106	-0.087
	(0.199)	(0.198)	(0.199)	(0.199)	(0.198)	(0.199)
Land × Drought	0.076	0.098	0.091	0.077	0.100	0.090
	(0.199)	(0.198)	(0.199)	(0.199)	(0.198)	(0.199)
Population	-0.084	-0.076	-0.078	-0.081	-0.073	-0.069
	(0.131)	(0.130)	(0.130)	(0.131)	(0.130)	(0.130)
Land × Population	0.060	0.050	0.093	0.071	0.061	0.089
	(0.139)	(0.138)	(0.135)	(0.139)	(0.138)	(0.135)
Ports	0.088***	0.087***	0.096***	0.089***	0.088***	0.097***
	(0.010)	(0.010)	(0.010)	(0.010)	(0.010)	(0.010)
Shipping	-0.003	-0.002	-0.008	-0.005	-0.005	-0.008
	(0.023)	(0.021)	(0.009)	(0.023)	(0.021)	(0.009)
Military base	-0.044***	-0.044***	-0.047***	-0.045***	-0.045***	-0.047***
	(0.010)	(0.010)	(0.010)	(0.010)	(0.010)	(0.010)
Total catch	0.177***	0.175***	0.099***			
	(0.022)	(0.022)	(0.012)			
Demersal catch				0.146***	0.145***	0.083***
				(0.023)	(0.022)	(0.013)
Pelagic catch				0.121***	0.119***	0.045***
				(0.024)	(0.024)	(0.013)
**W** × Fragility	-0.002	-0.032		-0.006	-0.034	
	(0.045)	(0.043)		(0.045)	(0.043)	
**W** × Luminosity	0.853**	0.674**		0.841**	0.671**	
	(0.270)	(0.250)		(0.271)	(0.251)	
**W** × Land	0.557	0.448		0.589	0.479	
	(0.438)	(0.401)		(0.437)	(0.401)	
**W** × Land × Luminosity	-1.069***	-0.884***		-1.061***	-0.885***	
	(0.278)	(0.257)		(0.279)	(0.259)	
**W** × Drought	1.630	1.329		1.648	1.347	
	(0.898)	(0.787)		(0.897)	(0.787)	
**W** × Land × Drought	-1.622	-1.317		-1.635	-1.331	
	(0.901)	(0.789)		(0.900)	(0.789)	

**W** × Population	-0.221	-0.158		-0.220	-0.163	
	(0.546)	(0.505)		(0.546)	(0.506)	
**W** × Land × Population	0.407	0.316		0.392	0.306	
	(0.553)	(0.512)		(0.553)	(0.512)	
**W** × Ports	-0.012	-0.028		-0.014	-0.030	
	(0.029)	(0.027)		(0.029)	(0.027)	
**W** × Shipping	0.007	0.002		0.010	0.006	
	(0.028)	(0.025)		(0.028)	(0.025)	
**W** × Military base	0.012	0.021		0.011	0.019	
	(0.028)	(0.026)		(0.028)	(0.026)	
**W** × Total catch	-0.165***	-0.176***				
	(0.037)	(0.035)				
**W** × Demersal catch				-0.133***	-0.143***	
				(0.039)	(0.037)	
**W** × Pelagic catch				-0.133***	-0.135***	
				(0.038)	(0.037)	
**W** × Piracy	0.544***	0.691***	0.547***	0.545***	0.690***	0.545***
	(0.015)	(0.019)	(0.015)	(0.015)	(0.019)	(0.015)
**W** × ϵ		-0.475***			-0.474***	
		(0.056)			(0.056)	
Wald ($p < \chi^2$)	0.000	0.000	0.000	0.000	0.000	0.000
Log(L)	-3.825×10^4	-3.822×10^4	-3.830×10^4	-3.824×10^4	-3.820×10^4	-3.829×10^4
R^2	0.053	0.053	0.039	0.054	0.054	0.040

Notes: Dependent variable is number of pirate incidents per maritime degree-cell during 2005-2009 and 2010-2014. Estimation is using maximum likelihood for spatially-weighted regression with random effects. Observations are restricted to degree-cells touching or within boundaries of exclusive economic zones (EEZs). Spatial-weighting matrix **W** is inverse-distance, spectrally normalized, and truncated to 250 km. All covariates are standardized. Intercepts and FAO fishing-area fixed effects are estimated but not reported. R^2 reported is McFadden's pseudo R^2. Standard errors robust to spatial autocorrelation and heteroskedasticity are in parentheses. $N = 22,676$. * $p < 0.10$; ** $p < 0.05$; *** $p < 0.01$.

S5 Table. Panel-spatial regressions: Robustness to larger radial truncation (exclusive economic zones only)

	500 km (1) – (3)			1,000 km (4) – (6)		
	**(1)**	**(2)**	**(3)**	**(4)**	**(5)**	**(6)**
Fragility	0.030<br>(0.027)	0.031<br>(0.027)	0.048***<br>(0.011)	0.067**<br>(0.021)	0.069**<br>(0.022)	0.053***<br>(0.011)
Luminosity	0.011<br>(0.075)	0.008<br>(0.075)	0.001<br>(0.073)	0.029<br>(0.075)	0.030<br>(0.075)	0.019<br>(0.074)
Land	-0.004<br>(0.110)	-0.004<br>(0.110)	0.023<br>(0.105)	-0.001<br>(0.110)	0.001<br>(0.110)	0.030<br>(0.106)
Land × Luminosity	0.144<br>(0.076)	0.147<br>(0.076)	0.087<br>(0.074)	0.130<br>(0.076)	0.129<br>(0.076)	0.080<br>(0.075)
Drought	-0.065<br>(0.201)	-0.069<br>(0.200)	-0.083<br>(0.201)	-0.026<br>(0.202)	-0.021<br>(0.203)	-0.067<br>(0.203)
Land × Drought	0.059<br>(0.200)	0.062<br>(0.200)	0.083<br>(0.201)	0.026<br>(0.202)	0.019<br>(0.202)	0.070<br>(0.203)
Population	-0.095<br>(0.132)	-0.093<br>(0.131)	-0.075<br>(0.131)	-0.064<br>(0.133)	-0.066<br>(0.133)	-0.071<br>(0.133)
Land × Population	0.028<br>(0.139)	0.025<br>(0.139)	0.058<br>(0.136)	0.022<br>(0.140)	0.023<br>(0.140)	0.044<br>(0.138)
Ports	0.091***<br>(0.010)	0.091***<br>(0.010)	0.099***<br>(0.010)	0.091***<br>(0.010)	0.090***<br>(0.010)	0.101***<br>(0.010)
Shipping	-0.001<br>(0.019)	-0.002<br>(0.018)	-0.010<br>(0.009)	-0.003<br>(0.016)	-0.002<br>(0.017)	-0.011<br>(0.010)
Military base	-0.046***<br>(0.010)	-0.046***<br>(0.010)	-0.049***<br>(0.010)	-0.050***<br>(0.010)	-0.050***<br>(0.010)	-0.051***<br>(0.010)
Total catch	0.180***<br>(0.019)	0.179***<br>(0.019)	0.102***<br>(0.013)	0.170***<br>(0.017)	0.171***<br>(0.017)	0.104***<br>(0.013)
**W** × Fragility	0.006<br>(0.044)	-0.002<br>(0.043)		-0.083<br>(0.044)	-0.082<br>(0.047)	
**W** × Luminosity	1.115*<br>(0.459)	1.011*<br>(0.445)		1.998*<br>(0.780)	2.263**<br>(0.837)	
**W** × Land	1.287<br>(0.769)	1.190<br>(0.740)		3.017*<br>(1.331)	3.286*<br>(1.421)	
**W** × Land × Luminosity	-1.435**<br>(0.476)	-1.323**<br>(0.461)		-2.517**<br>(0.810)	-2.803**<br>(0.870)	
**W** × Drought	2.904<br>(1.613)	2.631<br>(1.530)		5.388<br>(2.812)	6.251*<br>(3.075)	
**W** × Land × Drought	-2.891<br>(1.617)	-2.616<br>(1.533)		-5.396<br>(2.820)	-6.255*<br>(3.084)	
**W** × Population	-0.523<br>(0.962)	-0.508<br>(0.929)		-1.890<br>(1.677)	-1.828<br>(1.779)	

**W** × Land × Population	0.869	0.840		2.377	2.344	
	(0.970)	(0.937)		(1.688)	(1.790)	
**W** × Ports	-0.028	-0.034		-0.054	-0.046	
	(0.045)	(0.044)		(0.074)	(0.079)	
**W** × Shipping	0.007	0.007		0.016	0.016	
	(0.025)	(0.024)		(0.024)	(0.028)	
**W** × Military base	0.041	0.043		0.111	0.112	
	(0.043)	(0.041)		(0.070)	(0.074)	
**W** × Total catch	-0.257***	-0.257***		-0.339***	-0.347***	
	(0.044)	(0.043)		(0.056)	(0.060)	
**W** × Piracy	0.754***	0.800***	0.773***	0.889***	0.841***	0.920***
	(0.022)	(0.028)	(0.021)	(0.029)	(0.038)	(0.025)
**W** × ϵ		-0.189*			0.296**	
		(0.084)			(0.093)	
Wald ($p < \chi^2$)	0.000	0.000	0.000	0.000	0.000	0.000
Log(L)	-3.834×10^4	-3.834×10^4	-3.840×10^4	-3.849×10^4	-3.849×10^4	-3.855×10^4
R^2	0.059	0.059	0.034	0.061	0.061	0.022

Notes: Dependent variable is number of pirate incidents per maritime degree-cell during 2005-2009 and 2010-2014. Estimation is using maximum likelihood for spatially-weighted regression with random effects. Observations are restricted to degree-cells touching or within boundaries of exclusive economic zones (EEZs). Spatial-weighting matrix **W** is inverse-distance, spectrally normalized, and truncated to 500 km (columns 1 – 3) and 1,000 km (columns 4 – 6). All covariates are standardized. Intercepts and FAO fishing-area fixed effects are estimated but not reported. R^2 reported is McFadden's pseudo R^2. Standard errors robust to spatial autocorrelation and heteroskedasticity are in parentheses. $N = 22{,}676$. * $p < 0.10$; ** $p < 0.05$; *** $p < 0.01$.

S6 Table. Spatial instrumental-variables regressions

	Unrestricted (1) – (4)				Excluding W. Indian Ocean (5) – (8)			
	Full Sample		EEZs only		Full Sample		EEZs only	
	(1)	(2)	(3)	(4)	(5)	(6)	(7)	(8)
	2005 - 2009	2010 - 2014	2005 - 2009	2010 - 2014	2005 - 2009	2010 - 2014	2005 - 2009	2010 - 2014
IUU catch	0.234***	0.145***	0.225***	0.152***	0.208***	0.098***	0.215***	0.102***
	(0.035)	(0.018)	(0.048)	(0.025)	(0.035)	(0.017)	(0.049)	(0.023)
Fragility	0.080***	0.026***	0.079***	0.027**	0.059***	0.018***	0.059***	0.019*
	(0.013)	(0.006)	(0.018)	(0.009)	(0.013)	(0.006)	(0.018)	(0.008)
Luminosity	-0.189	-0.098	-0.190	-0.093	-0.167	-0.016	-0.187	-0.014
	(0.128)	(0.084)	(0.177)	(0.115)	(0.151)	(0.093)	(0.211)	(0.129)
Land	-0.147*	-0.001	-0.101	0.018	-0.137*	-0.025	-0.091	-0.002
	(0.066)	(0.042)	(0.093)	(0.059)	(0.068)	(0.039)	(0.096)	(0.056)
Land × Luminosity	0.341**	0.189*	0.354*	0.193	0.351*	0.122	0.388	0.129
	(0.130)	(0.086)	(0.180)	(0.117)	(0.154)	(0.094)	(0.215)	(0.131)
Drought	0.070	-0.020	-0.079	0.007	0.116	0.008	-0.061	0.025
	(0.352)	(0.245)	(0.492)	(0.338)	(0.441)	(0.303)	(0.624)	(0.426)
Land × Drought	-0.064	-0.005	0.084	-0.029	-0.075	-0.04	0.105	-0.057
	(0.353)	(0.245)	(0.494)	(0.339)	(0.442)	(0.303)	(0.625)	(0.427)
Population	0.002	-0.013	-0.012	-0.011	0.012	-0.013	-0.005	-0.010
	(0.024)	(0.015)	(0.034)	(0.021)	(0.025)	(0.014)	(0.037)	(0.020)
Land × Population	-0.002	-0.002	0.010	-0.006	-0.011	0.002	0.003	-0.003
	(0.025)	(0.015)	(0.035)	(0.022)	(0.026)	(0.014)	(0.038)	(0.021)
Ports	0.226***	0.107***	0.223***	0.106***	0.191***	0.113***	0.185***	0.111***
	(0.019)	(0.012)	(0.027)	(0.017)	(0.019)	(0.011)	(0.027)	(0.016)
Shipping	0.038*	-0.006	0.057	-0.007	-0.001	-0.005	-0.002	-0.005
	(0.017)	(0.004)	(0.031)	(0.008)	(0.017)	(0.004)	(0.031)	(0.007)
Military bases	-0.139***	-0.053***	-0.134***	-0.051**	-0.127***	-0.054***	-0.122***	-0.052**
	(0.020)	(0.013)	(0.028)	(0.018)	(0.020)	(0.012)	(0.028)	(0.017)
**W** × Piracy	0.545***	0.819***	0.596***	0.806***	0.555***	0.843***	0.612***	0.850***
	(0.083)	(0.071)	(0.105)	(0.092)	(0.092)	(0.072)	(0.116)	(0.091)
N	21,836	21,836	11,338	11,338	19,296	19,296	9,814	9,814
Wald ($p < \chi^2$)	0.000	0.000	0.000	0.000	0.000	0.000	0.000	0.000
R^2	0.044	0.032	0.044	0.036	0.040	0.028	0.410	0.034

Notes: Dependent variable is number of pirate incidents per maritime degree-cell during 2005-2009 and 2010-2014. Estimation is by generalized spatial two-stage least. Illegal, unreported, and unregulated (IUU) fish catch is instrumented with ocean net primary production (NPP) for that degree-cell. For EEZ-only subsamples, observations are restricted to degree-cells touching or within boundaries of exclusive economic zones (EEZs). Spatial-weighting matrix **W** is inverse-distance, spectrally normalized, and truncated to 250 km. Instrument matrix includes zero- and first-order spatial lags of all instruments. Intercepts and FAO fishing-area fixed effects are estimated but not reported. R^2 reported is McFadden's pseudo R^2. Standard errors robust to spatial autocorrelation and heteroskedasticity are in parentheses. $N = 22,676$. * $p < 0.10$; ** $p < 0.05$; *** $p < 0.01$.

References

Behrenfeld, M. J., and Falkowski, P. G. (1997). Photosynthetic rates derived from satellite-based chlorophyll concentration. *Limnology and Oceanography* 42(1): 1-20.

Halpern, B.S., Frazier, M., Potapenko, J., Casey, K.S., Koenig, K., Longo, C., Lowndes, J.S., Rockwood, R.C., Selig, E.R., Selkoe, K.A. and Walbridge, S. (2015). Spatial and temporal changes in cumulative human impacts on the world's ocean. *Nature* 6(1): 1-7.

Halpern, B.S., Walbridge, S., Selkoe, K.A., Kappel, C.V., Micheli, F., D'Agrosa, C., Bruno, J.F., Casey, K.S., Ebert, C., Fox, H.E. and Fujita, R. (2008). A global map of human impact on marine ecosystems. *Science* 319(5865): 948-952.

Organisation for Economic Cooperation and Development [OECD] (2014). *Fragile states 2014: Domestic revenue mobilisation in fragile states*. Paris, OECD.

——— (2013). *Fragile states 2013: Resource flows and trends in a shifting world*. Paris, OECD.

——— (2012). *Conflict and fragility: Managing risks in fragile and transitional contexts*. Paris, OECD.

——— (2011). *2011 report on financial resource flows: Ensuring fragile states are not left behind*. Paris, OECD.

——— (2010). *Resource flows to fragile and conflict-affected states*. Paris, OECD.

——— (2009). *Ensuring fragile states are not left behind*. Paris, OECD.

——— (2008). *Monitoring resource flows to fragile and conflict-affected states*. Paris, OECD.

——— (2007). *Monitoring resource flows to fragile states*. Paris, OECD.

——— (2006). *Monitoring resource flows to fragile states*. Paris, OECD.

——— (2005). *Monitoring resource flows to fragile states*. Paris, OECD.

Rogers J, Simon L. (2009). The status and location of the military installations of the member states of the European Union and their potential role for the European security and defence Policy (ESDP). Directorate-General for External Policies of the Union Briefing Paper 19, European Parliament, Brussels.

Vine, D. (2015). *Base nation: How US military bases abroad harm America and the world*. Metropolitan Books.
